# Supplementary material for: DMRforPairs: identifying Differentially Methylated Regions between unique samples using array based methylation profiles
Source: BMC Bioinformatics. 2014 May 15;15:141. doi: 10.1186/1471-2105-15-141 (PMC4046028; doi:10.1186/1471-2105-15-141)
Supplement: Additional file 2 — DMRforPairs output for the comparison of A431-MCF7 and NA17018-NA17105. Please start from the HTML files in each folder. Available via the BMC Bioinformatics website. [file 1471-2105-15-141-S2.zip › 1394847754114233_MOESM2_ESM/A431_MCF7/figures/2868.pdf]

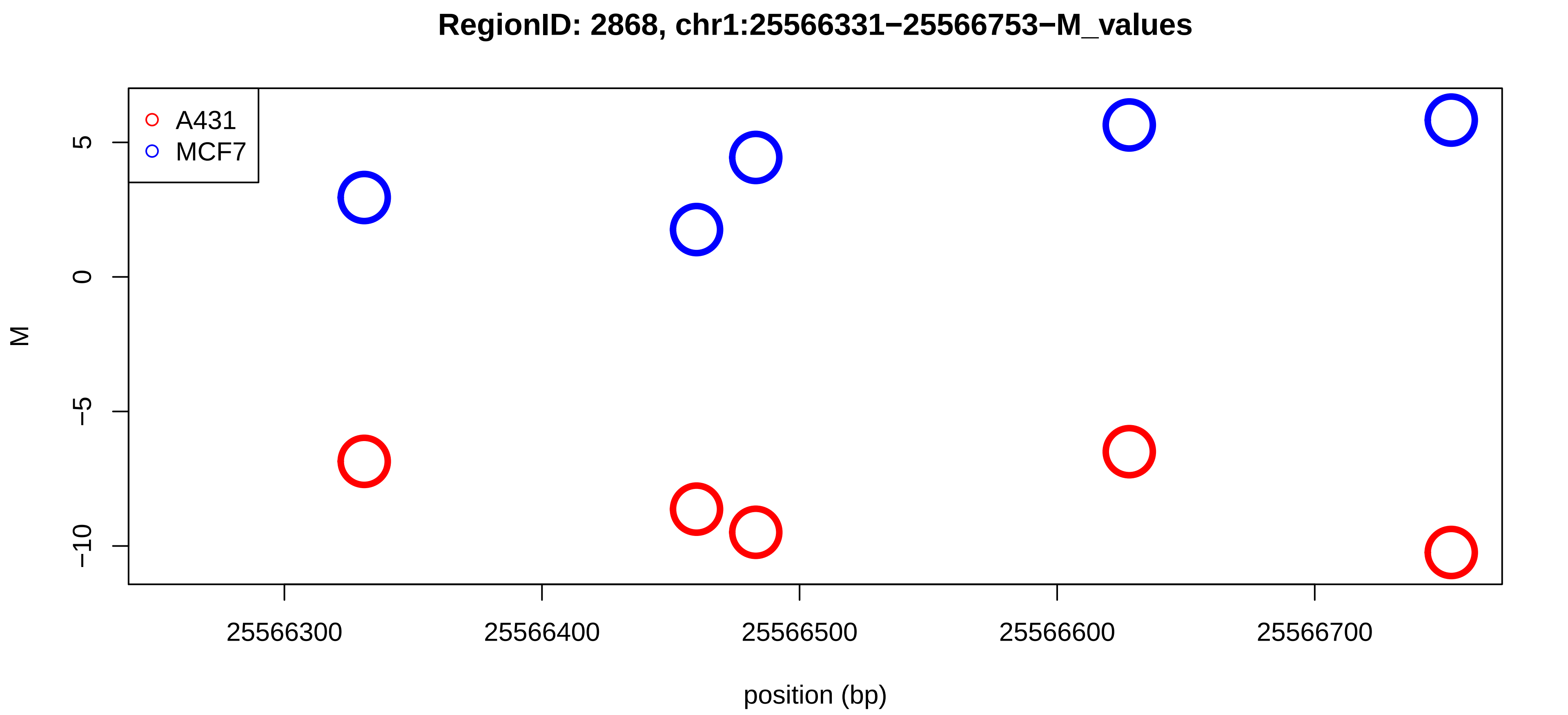
RegionID: 2868, chr1:25566331−25566753−M_values
A431
MCF7
5
0
−5
−10
M
25566300
25566400
25566500
25566600
25566700
position (bp)

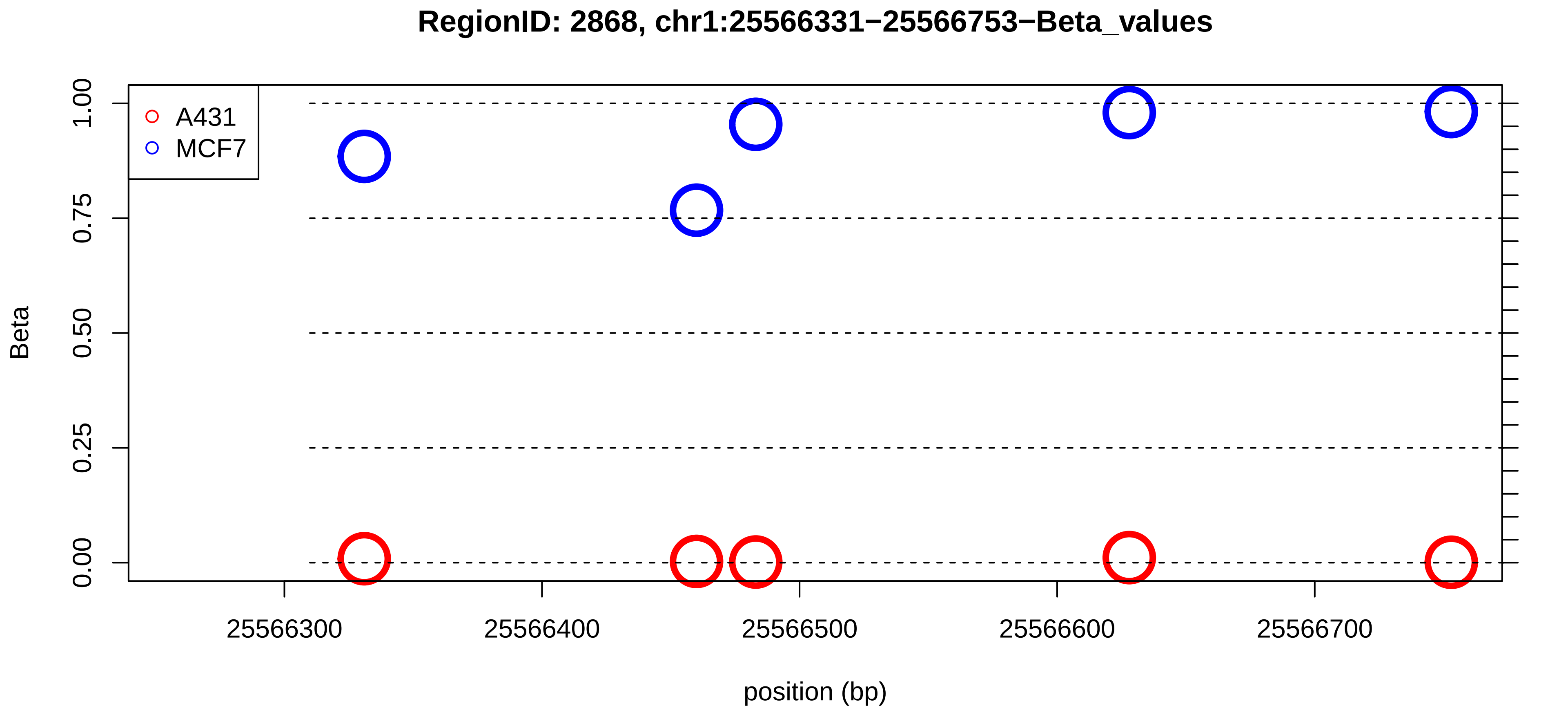
RegionID: 2868, chr1:25566331−25566753−Beta_values
A431
MCF7
1.00
0.75
0.50
0.25
0.00
Beta
25566300
25566400
25566500
25566600
25566700
position (bp)

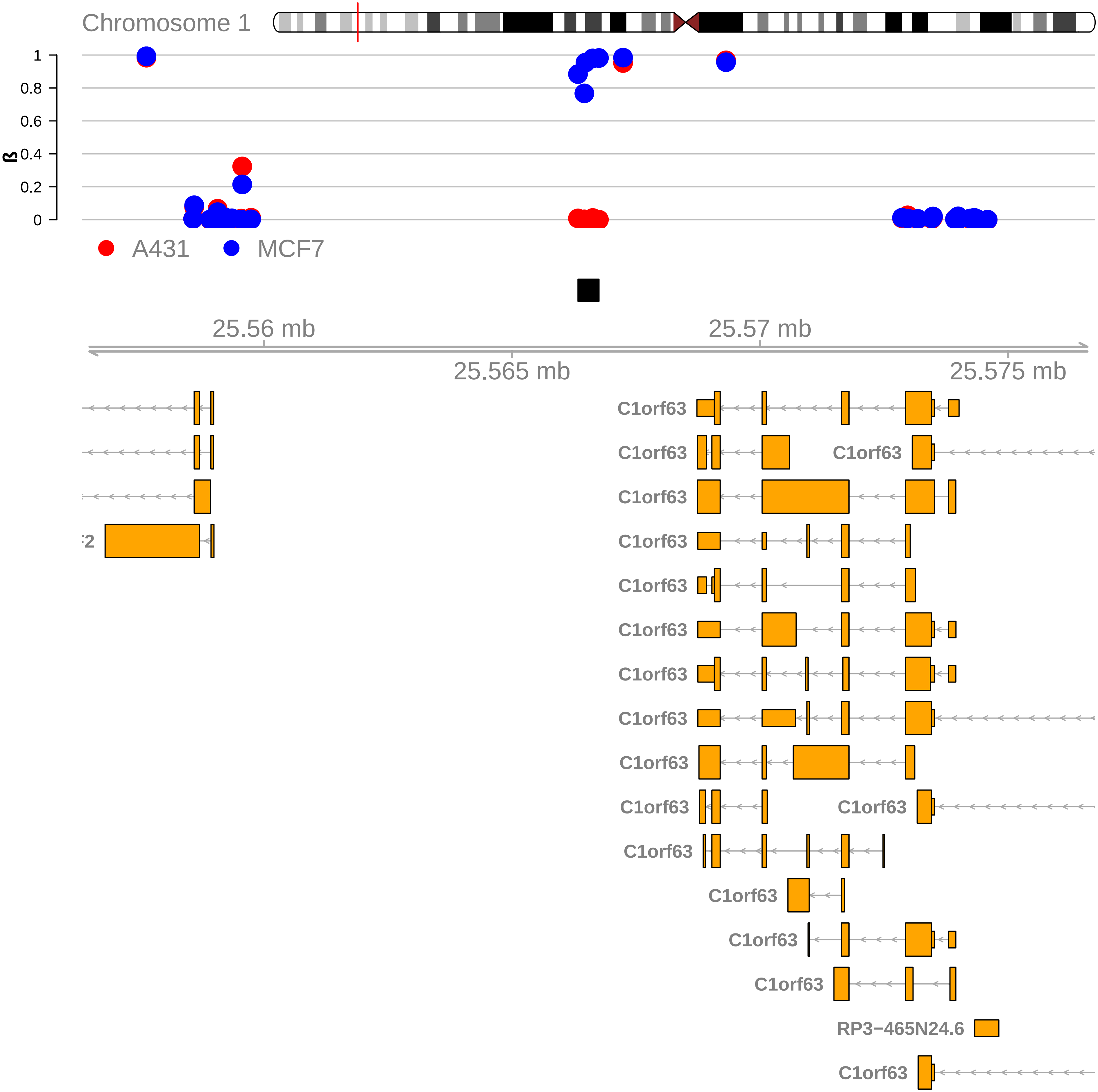

Chromosome 1
ß
1
0.8
0.6
0.4
0.2
0
A431
MCF7
25.56 mb
25.565 mb
25.57 mb
25.575 mb
C1orf63
RP3−465N24.6
